# CGRP-induced migraine-like headache in persistent post-traumatic headache attributed to mild traumatic brain injury

**DOI:** 10.1186/s10194-022-01499-5

**Published:** 2022-10-17

**Authors:** Håkan Ashina, Afrim Iljazi, Haidar M. Al-Khazali, Thien Phu Do, Anna K. Eigenbrodt, Eigil L. Larsen, Amalie M. Andersen, Kevin J. Hansen, Karoline B. Bräuner, Basit Ali Chaudhry, Casper E. Christensen, Faisal Mohammad Amin, Henrik W. Schytz

**Affiliations:** 1 Department of Anesthesia, Critical Care and Pain Medicine, Beth Israel Deaconess Medical Center, Harvard Medical School, Boston, MA USA; 2 Danish Headache Center, Department of Neurology, Faculty of Health and Medical Sciences, Rigshospitalet – Glostrup, University of Copenhagen, Copenhagen, Denmark; 3 Department of Neurorehabilitation / Traumatic Brain Injury, Rigshospitalet, Copenhagen, Denmark

**Keywords:** Pathophysiology, Trigeminovascular System, Concussion, Migraine, CGRP

## Abstract

**Objective:**

To ascertain whether intravenous infusion of calcitonin gene-related peptide (CGRP) can induce migraine-like headache in people with persistent post-traumatic headache attributed to mild traumatic brain injury (TBI) and no pre-existing migraine.

**Methods:**

A non-randomized, single-arm, open-label study at a single site in Denmark. Eligible participants were aged 18 to 65 years and had a known history of persistent post-traumatic headache attributed to mild TBI for ≥ 12 months. All participants received continuous intravenous infusion of CGRP (1.5 µg/min) over 20 min. A headache diary was used to collect outcome data until 12 h after the start of CGRP infusion. The primary end point was the incidence of migraine-like headache during 12-hour observational period.

**Results:**

A total of 60 participants completed the study protocol and provided data for the analysis of the primary end point. The median age was 32.5 (IQR, 25.5–43.0) years; 43 participants (72%) were female. Following CGRP infusion, 43 (72%) of 60 participants developed migraine-like headache during the 12-hour observational period. The median time to peak headache intensity was 40 min (IQR, 20–60), and the median peak headache intensity was 6 (IQR, 5–8) on the 11-point numeric rating scale.

**Conclusion:**

Intravenous infusion of CGRP is a potent inducer of migraine-like headache in people with persistent post-traumatic headache attributed to mild TBI. This observation underscores the importance of CGRP in the genesis of migraine-like headache that is often experienced by individuals who are afflicted by persistent post-traumatic headache. Further research is warranted to ascertain whether other signaling molecules also contribute to the disease mechanisms underlying post-traumatic headache.

## Introduction

Post-traumatic headache is a disabling neurologic disorder that remains a challenge for clinicians and researchers alike [1]. Although most people recover from acute post-traumatic headache within three months of onset following traumatic injury to the head [2–4], some experience persistence of cephalic pain beyond the 3-month mark and are then diagnosed with persistent post-traumatic headache [5–7]. The latter is often associated with exacerbations of cephalic pain that resemble the clinical features of migraine. The frequency of these exacerbations varies among patients [8], and little is known about the underlying molecular signaling mechanisms [9]. However, increased attention is being paid to the possible involvement of the signaling molecule calcitonin gene-related peptide (CGRP) [10]. A recent randomized, double-blind, placebo-controlled trial found that intravenous infusion of CGRP induced migraine-like headache in 21 (70%) of 30 participants with persistent post-traumatic headache, compared with 6 (20%) of 30 participants after placebo infusion [11]. This finding provides compelling evidence of CGRP involvement in the genesis of migraine-like headache among those with persistent post-traumatic headache [12]. However, the results must be replicated in a larger sample before firm conclusions can be drawn.

In this non-randomized, single-arm, open-label study, we seek to confirm that intravenous infusion of CGRP induces migraine-like headache in most people with persistent post-traumatic headache attributed to mild traumatic brain injury (TBI).

## Methods

### Study oversight

The study protocol was approved by the relevant ethics committee and data protection agency. All participants provided written informed consent before any protocol-related procedures or assessments were performed. The study was conducted in accordance with the principles of the Declaration of Helsinki.

### Study population

Eligible participants were aged 18 to 65 years and had a known history of persistent post-traumatic headache attributed to mild TBI for ≥ 12 months that also met the diagnostic criteria outlined in the International Classification of Headache Disorders, 3rd edition (ICHD-3) [13]. Key exclusion criteria were any known history of > 1 TBI or whiplash injury. Potential participants were also excluded if they had any history of a primary headache disorder, except for infrequent episodic tension-type headache (TTH). Electronic medical records were reviewed to ensure eligibility for study inclusion.

### Study design

Participants were enrolled in a non-randomized, single-arm, open-label study that was conducted at the Danish Headache Center. All received continuous intravenous infusion of CGRP (1.5 µg/min) in the antecubital fossa over 20 min using a time- and volume-controlled infusion pump.

The CGRP dose is identical to the one used in previous experimental studies that included people with persistent post-traumatic headache, migraine, and cluster headache [11, 14–16]. Independent hospital pharmacy staff were responsible for drug preparation. Participants were told that CGRP might induce headache; no information was provided on its possible onset, clinical features, or accompanying symptoms.

On the experimental day, participants were scheduled to arrive between 08:00 AM and 13:00 PM. A site investigator would then perform a semi-structured interview to collect data on demographics, medical history, and full clinical course. Following this, participants underwent a physical and neurologic examination, in addition to a 12-lead electrocardiogram. Eligibility on the experimental day was contingent upon the participant reporting no intake of acute medications (e.g., simple analgesics, triptans) within 48 h of infusion start and a baseline headache intensity of ≤ 5 on an 11-point numeric rating scale (NRS, 0 being no headache, 10 being the worst imaginable headache). Furthermore, it was required that participants did not report, if relevant, their usual migraine-like headache at baseline, with the latter defined as the time of infusion start (T_0_).

Participants were placed in a supine position at the time of infusion start and during the in-hospital phase (0–90 min), a site investigator would collect data on headache characteristics, use of rescue medication, adverse events, and hemodynamics (i.e., blood pressure and heart rate) every 10 min until 60 min after the start of infusion and then again at 90 min after infusion start. Hereafter, participants would be discharged with a headache diary that they needed to fill out hourly until 12 h after infusion start.

### Statistical analysis

Descriptive statistics were used to summarize demographic and clinical characteristics, and the Shapiro-Wilk test was performed to evaluate whether the variables followed a normal distribution. The median value with interquartile ranges (IQR) or the mean with standard deviations (SD) were then used as appropriate. The primary end point was the incidence of migraine-like headache during the 12-hour observational period after the start of CGRP infusion. Predefined criteria were used to assess whether the participants developed migraine-like headache during the 12-hour observational period (Table 1). Secondary end points included median time to peak headache intensity, median peak headache intensity, and baseline corrected area under the curve (AUC) for headache intensity scores (0–12 h). The sample size was based on the number of individuals who participated in a clinical trial and were also interested in undergoing provocation with CGRP in the present study. All analyses were performed with R (v4.1.2).

**Table 1:** Criteria for Experimentally-Induced Migraine-Like Headache in People with Persistent Post-Traumatic Headache

**Migraine-like headache has at least two of the following characteristics**: • Unilateral location, • Pulsating quality, • Moderate to severe pain intensity, and • Aggravation by or causing avoidance of routine physical activity (e.g., walking or climbing stairs);
**And during headache, at least one of the following**: • Nausea and/or vomiting, • Photophobia and phonophobia, or • Headache mimicking their usual migraine-like headache

## Results

### Characteristics of the Study Population

**Fig. 1:**
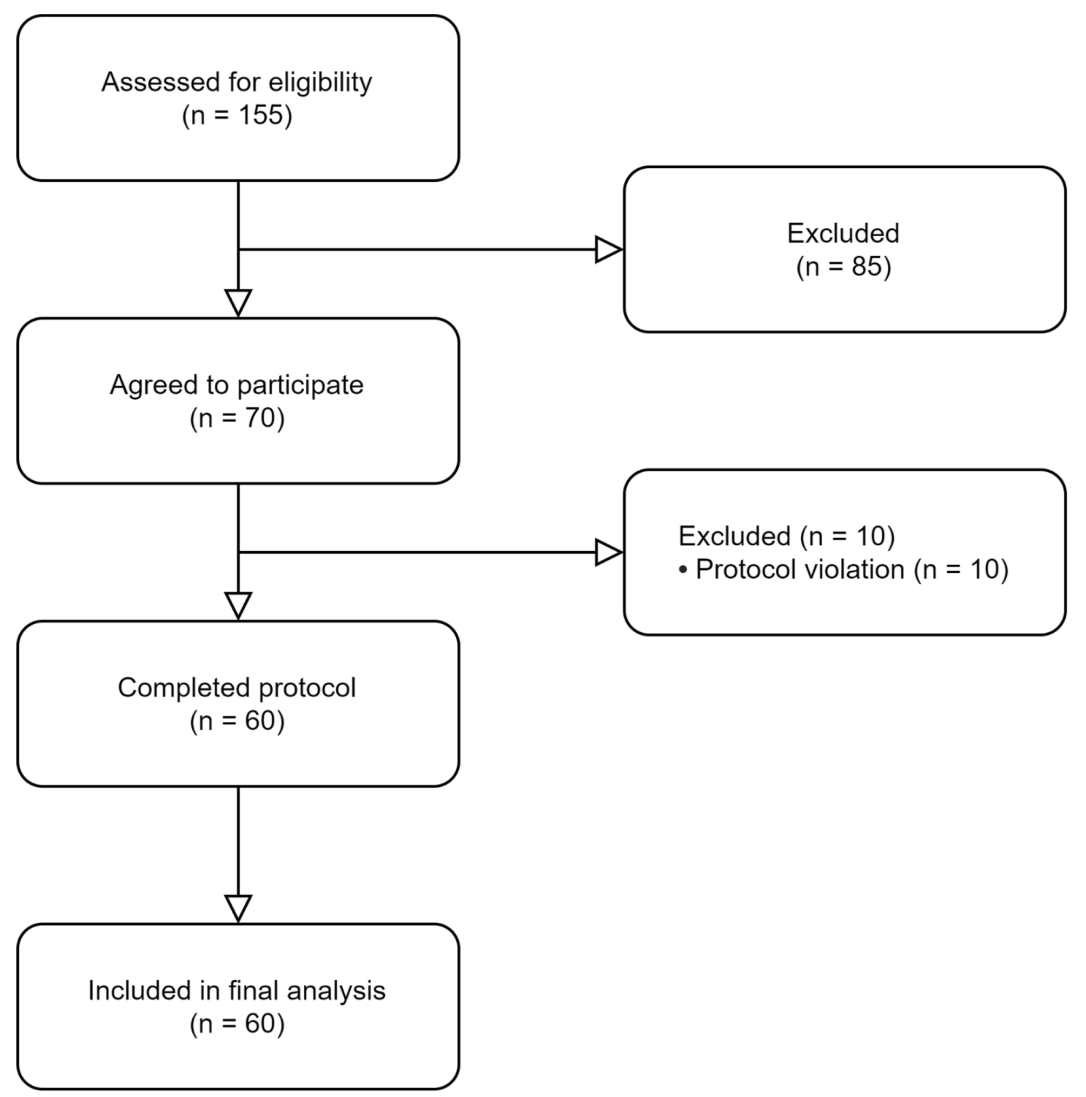
Recruitment Flowchart of Participants with Persistent Post-Traumatic Headache

The study included 60 participants who received intravenous infusion of CGRP and provided data for the analysis of the primary end point (Fig. 1; Table 2). The median age was 32.5 (IQR, 25.5–43.0) years; 43 participants (72%) were female, and 21 participants (35%) reported current use of preventive medications. In addition, the median number of monthly headache days was 28 (IQR, 23.0–28.0), while the median number of monthly headache days of moderate to severe intensity was 13.5 (IQR, 4.8–23.3). Most participants (52 [87%]) had a migraine-like phenotype while a ‘pure’ TTH-like phenotype was less common (8 [13%]).

**Table 2:** Characteristics of the Study Population

Study Population Characteristics	All Participants (n = 60)
**Age, median (IQR)**	32.5 (25.5–43.0)
**BMI, mean (SD)**	25.9 (5.9)
**Weight, mean (SD)**	77.6 (18.9)
**Men/Women, n (%)**	17/43 (28% / 72%)
**Employment Status**	
• Full-Time, n (%) • Part-Time, n (%) • Unemployed, n (%) • Retired, n (%)	• 26 (43%) • 23 (38%) • 10 (17%) • 1 (2%)
**Years of Education, mean (SD)**	14.1 (2.7)
**Education**	
• No Education (aside from completion or secondary school or high school), n (%) • Skilled Labour, n (%) • Bachelor’s Degree, n (%) • Higher Education, n (%)	• 10 (17%) • 15 (25%) • 18 (30%) • 17 (28%)
**Family History of Primary Headache Disorder, n (%)**	23 (38%)
**Headache Phenotype**	o
• Migraine-Like, n (%) o Episodic Migraine-Like, n (%) o Episodic Migraine-Like with Frequent Tension-Type Headache-Like, n (%) o Episodic Migraine-Like Combined with Chronic Tension-Type Headache-Like, n (%) o Chronic Migraine-Like, n (%) • Tension-Type Headache Like, n (%) o Episodic Tension-Type Headache-Like, n (%) o Chronic Tension-Type Headache-Like, n (%)	• 52 (87%) o 2 (3%) o 3 (5%) o 12 (20%) o 35 (58%) • 8 (13%) o 0 (0%) o 8 (13%)
**Injury Cause**	
• Fall, n (%) • Motor Vehicle Collision, n (%) • Sports-Related Injury, n (%) • Violence/Assault, n (%) • Other Unintentional Injury, n (%)	• 16 (27%) • 16 (27%) • 11 (18%) • 4 (7%) • 13 (22%)
^**a**^ **Time (No. of Months) since Mild TBI, median (IQR)**	48.0 (26.0–63.0)
^**b**^ **Self-Rated Health, median (IQR)**	3.0 (3.0–4.0)
**History of Comorbid Psychiatric Disorder, n (%)**	11 (18%)
^**a**^ **Number of Monthly Headache Days, median (IQR)**	28 (23.0–28.0)
^**a**^ **Number of Monthly Headache Days of Moderate to Severe Headache Intensity, median (IQR)**	13.5 (4.8–23.3)
**Satisfied with Current Treatment Status, n (%)**	18 (30%)
**Current Use of Preventive Medication, n (%)**	21 (35%)
**History Use of Preventive Medication, n (%)**	45 (75%)
**Number of Prior Failed Preventive Medications, n (%)**	
• 0 • 1 • 2 • 3 • 4 or more • N/A	• 9 (15%) • 12 (20%) • 11 (18%) • 6 (10%) • 7 (12%) • 15 (25%)
**Current Use of Acute Medication, n (%)**	53 (88%)
**Number of Acute Medication Intake Days, median (IQR)**	4.0 (0.0–7.0)

Abbreviations: BMI, body mass index; No., number; SD, standard deviation

^a^ Retrospectively collected at baseline by semi-structured interview

^b^ Rated on a 5-point Likert scale (1: poor, 2: rather poor, 3: good, 4: great, 5: excellent)

#### Migraine-like headache

**Fig. 2:**
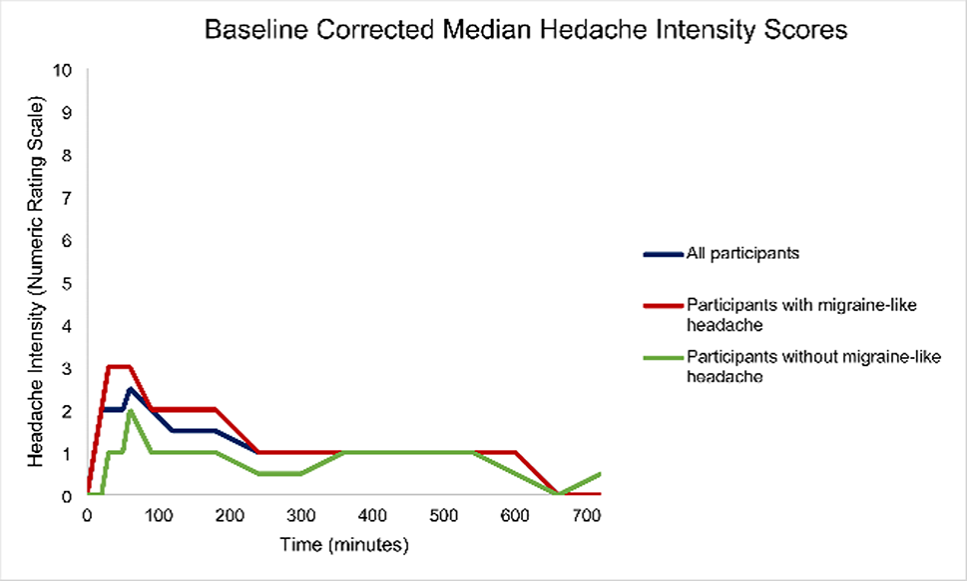
Baseline-corrected median headache intensity scores during the 12-hour (i.e. 720 min) observational period after intravenous infusion of calcitonin gene-related peptide (thick lines). Median time to peak headache intensity was 40.0 (IQR, 20.0–60.0) minutes

Intravenous infusion of CGRP induced migraine-like headache in 43 (72%) of 60 participants during the 12-hour observational period (Supplemental File 1). The median time to peak headache intensity was 40 min (IQR, 20–60), and the median peak headache intensity was 6 (IQR, 5–8) on NRS among all study participants (Fig. 2). Furthermore, migraine-like headache was experienced by 39 (75%) of 52 participants who had a migraine-like phenotype, while 4 (50%) of 8 participants with a ‘pure’ TTH-like phenotype reported migraine-like headache. Thirteen (22%) of 60 participants took rescue medication during the 12-observational period.

Among those who developed a migraine-like headache (n = 43), the median time to peak headache intensity was 40 min (IQR, 20–60), and the median peak headache intensity was 7 (IQR, 6–8) on NRS. Most (n = 38) reported migraine-like headache within 90 min after the start of infusion. Moreover, migraine-like headache was reported by 19 (90%) of 21 participants who had current use of preventive medications and 24 (62%) of the remaining 39 participants who did not use preventive medications at the time of study inclusion.

#### Adverse events and hemodynamics

**Fig. 3:**
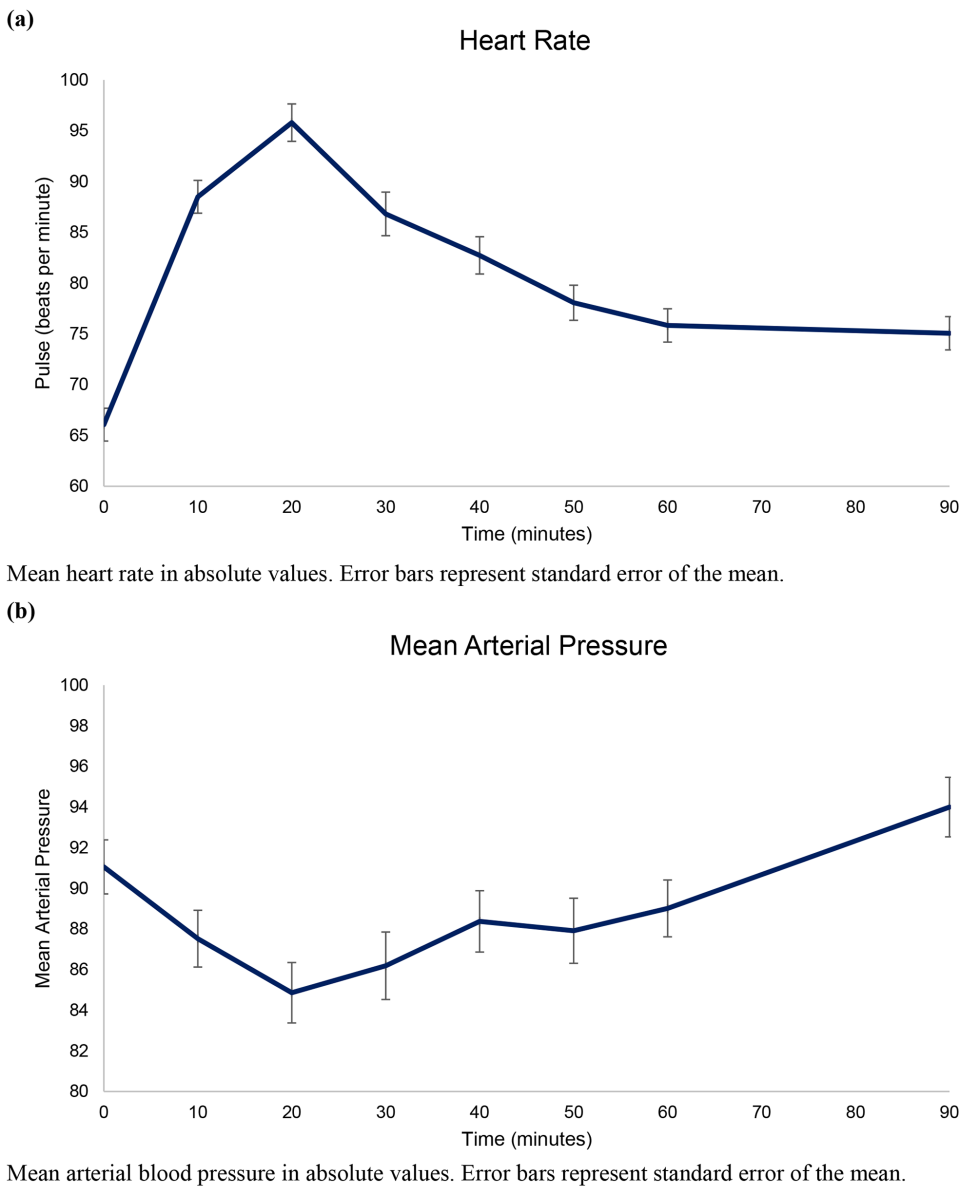
Mean Heart Rate (a) and Mean Arterial Blood Pressure (b) during the 90-Minute In-Hospital Observational Period

The most common adverse event was flushing (n = 59) followed by warm sensations (n = 58) and palpitations (n = 42). Less common adverse events were abdominal discomfort (n = 3), tiredness (n = 2), increased appetite (n = 2), and tension in the jaw (n = 2). In terms of hemodynamics, mean arterial blood pressure decreased while the heart rate increased during the 90-minute in-hospital phase after the start of infusion (Fig. 3a and b).

## Discussion

In this open-label study of patients with persistent post-traumatic headache, 43 (72%) of 60 participants developed migraine-like headache during the 12-hour observational period after intravenous infusion of CGRP. This finding accords well with a recent randomized, double-blind, placebo-controlled trial in which the 21 (70%) of 30 participants with persistent post-traumatic headache developed migraine-like headache after CGRP infusion [11]. The similar induction rates of migraine-like headache also suggest that there are limited to no nocebo effects in provocation experiments with CGRP in patients with persistent post-traumatic headache. However, the median time to peak headache intensity was 40 min after the start of CGRP infusion in the present study, compared with 120 min in the aforementioned randomized, double-blind, placebo-controlled trial [11]. It remains unclear whether the more rapid time to peak headache intensity is, in part, attributable to nocebo effects, which could also the higher peak headache intensity in the present study, compared with placebo-controlled trials.

The site and mechanism of action by which CGRP induces migraine-like headache is incompletely delineated [17–20], albeit multiple animal studies have established that CGRP can modulate nociceptive transmission in concussed rodents [21–24]. A compelling theory has recently been posited to explain migraine pathogenesis and might also extend, in part, to the genesis of migraine-like headache in people with post-traumatic headache [17]. This theory suggests that CGRP binds to its G protein-coupled receptor on the vascular smooth muscle cell within the walls of intracranial arteries and then initiates downstream intracellular signaling resulting in the opening of specific potassium channels. The latter causes efflux of potassium and accompanying vasodilation [17, 25–27]. This, in turn, is thought to provide chemical and mechanical stimuli sufficient to activate and sensitize perivascular nociceptors that project to first-order neurons in the trigeminal ganglion and upper cervical dorsal root ganglia [17]. From here, ascending trigeminal pain pathways relay the nociceptive information to cortical and subcortical regions that are responsible for the perception of migraine-like pain. In support of this theory, ample experimental data have shown that dilators of intracranial arteries (including specific potassium channel openers) can induce migraine attacks in people with migraine [26–34]. However, further studies are needed to draw firm conclusions on the link between dilation of intracranial arteries and subsequent activation of perivascular nociceptors in the genesis of migraine-like headache.

Another possible explanation is that CGRP elicits migraine-like headache through direct binding to its receptors on primary afferents that originate from the trigeminal ganglion [35]. Increased attention is being paid to this line of reasoning because animal data have shown that fremanezumab – a monoclonal antibody (mAb) against CGRP – inhibits nociceptive transmission from meningeal nociceptors by decreasing their responsiveness [36]. The same lab also reported that injection of fluorescently-labeled fremanezumab in rodents can be detected in the meninges and its blood vessels as well as in the sensory and autonomic ganglia [37]. However, no fluorescently-labeled fremanezumab was observed within CNS structures, including the brain stem, thalamus, hypothalamus, and cortex. Although these findings do not exclude involvement of the intracranial vasculature in the genesis of migraine-like headache, it seems reasonable to further explore whether CGRP mediates its pro-nociceptive effects via direct receptor-binding on primary afferents of the trigeminal ganglion and (upper cervical dorsal root ganglia). It should also be noted that some intriguing evidence suggest that CGRP-mediated nociceptive signals might be mediated via Schwann cells [38].

The involvement of CGRP in the mechanisms underlying post-traumatic headache is a rapidly expanding field. Recent open-label trial data also suggest that erenumab – a mAb against the CGRP receptor – holds promise for the preventive treatment of persistent post-traumatic headache [39]. However, it is unlikely that CGRP is the only pathogenic driver of migraine-like headache in people afflicted by persistent post-traumatic headache. There is a clear need to study responses to other signaling molecules that are well-established modulators of nociceptive transmission in migraine, e.g. adrenomedullin, amylin, and pituitary adenylate cyclase-activating polypeptide [31, 32, 40]. Establishing the contribution of possible pathogenic drivers should facilitate the identification of novel drug targets which, in turn, can be applied for patient benefit. Concerted efforts are also needed to clarify the pathogenic drivers involved in disease persistence and chronification of post-traumatic headache [9, 41, 42]. The most obvious unanswered scientific question remains why post-traumatic headache remits in some people and persists in others. In addition, the phenotypic and pathophysiologic similarities between post-traumatic headache and migraine should capture more interest, as it remains unclear whether head trauma can evoke migraine in susceptible individuals and perhaps even chronic migraine in those who previously had episodic migraine. If the latter is proven to be true, head trauma would constitute a risk factor for migraine chronification.

### Limitations

This study has several limitations. First, some participants reported current use of preventive medications which might affect the likelihood of developing migraine-like headache after CGRP infusion. It would be interesting if larger studies are performed to examine whether the use of specific preventive medications is associated with a lower induction rate of migraine-like headache. Second, the in-hospital phase lasted until 90 min after the start of infusion. Patient were then discharged for the remaining observational period, and it is therefore not possible to exclude the influence of environmental factors (e.g. stress, specific foods). However, it should be noted that 38 (88%) of 43 participants who developed migraine-like headache did so during the in-hospital phase. Third, a prospective headache diary with daily entries was not used to record the number of migraine-like days in the preceding month. It would be useful if future studies investigated whether the occurrence of CGRP-induced migraine-like headache is related to the frequency of migraine-like headache in the preceding month or time since the last episode with migraine-like headache. Lastly, the study population was limited to people with persistent post-traumatic headache, and our findings can therefore not be extended to conclude on the possible involvement of CGRP in acute post-traumatic headache.

## Conclusion

Among participants with persistent post-traumatic headache attributed to mild TBI, most developed migraine-like headache during the 12-hour observational period following intravenous infusion of CGRP. Thus, it seems evident that CGRP is an important signaling molecule in the pathogenesis of persistent post-traumatic headache. The involvement of other signaling molecules should be investigated in future studies to discover pathogenic drivers of post-traumatic headache.

## Data Availability

Qualified researchers can request access to patient-level data and related study documents, including the study protocol. Patient-level data will be deidentified and study documents will be redacted to protect the privacy of study participants.

## Abbreviations

CGRP: Calcitonin gene-related peptide.
TBI: Traumatic brain injury.
ICHD-3: International Classification of Headache Disorders, 3rd edition.
TTH: Tension-type headache.
IQR: Interquartile range.
SD: Standard deviations.
AUC: Area under the curve.
mAb: Monoclonal antibody.
